# Interlaminar endoscopic discectomy for recurrent L4–5 and L5–S1 disc herniation

**DOI:** 10.7717/peerj.21081

**Published:** 2026-04-27

**Authors:** Yongsheng Ye, Yonghong Feng, Cailiang Cai, Xuefei Zhang, Henian Li, Binshan Zhang, Jianqiu Chen, Shabin Zhuang, Fangyue Deng

**Affiliations:** Department of Orthopedics, Dongguan Hospital of Traditional Chinese Medicine, Dongguan, Guangdong Province, China

**Keywords:** Discectomy, Disc herniation, Recurrent lumbar disc herniation, Revision, Lumbar, Percutaneous endoscopic interlaminar discectomy

## Abstract

**Background:**

Revision for recurrent lumbar disc herniation (RLDH) is associated with epidural scarring and postoperative segmental instability. Endoscopic surgery is associated with better clarity, organised identification, fewer tissue lesions, and quicker recovery than open revision. We aimed to evaluate the effectiveness of percutaneous endoscopic interlaminar discectomy (PEID) for patients with RLDH at L4–5 and L5–S1.

**Methods:**

A total of 64 consecutive patients with RLDH underwent full endoscopic interlaminar discectomy between June 2022 and December 2024. Clinical outcomes were measured *via* the visual analogue scale (VAS) for back and leg pain, the Oswestry Disability Index (ODI), and the modified MacNab criteria through a 2-year follow-up period.

**Results:**

Significant improvements in VAS for back and leg pain and the ODI were observed post-treatment. Based on the modified MacNab criteria, 56 out of 64 patients demonstrated excellent and good, four cases in fair and four cases in poor at the 2-year follow-up.

**Conclusion:**

PEID yields favourable clinical outcomes in patients with RLDH at L4–5 and L5–S1 within this cohort.

## Introduction

Recurrent lumbar disc herniation (RLDH) is the recurrence of disc herniation in the same segment operated on at least 6 months prior. The recurrence rate ranges from 5% to 18%, depending on the surgical approach (Suk et al., 2001; Shriver et al., 2015). Standard treatments for RLDH include repeated lumbar discectomy for patients with spinal stability and instrumented fusion for those with spinal instability. Regarding repeat discectomy, increasing attention has been given to endoscopic discectomy for RLDH, predominantly through a posterolateral approach (Hoogland et al., 2008; Gadjradj et al., 2022). Percutaneous endoscopic transforaminal discectomy (PETD) through unscarred tissue can alleviate nerve injury and damage to posterior spinal structures (Hoogland et al., 2008; Gadjradj et al., 2022).

With recent advancements in percutaneous endoscopy, including PETD and percutaneous endoscopic interlaminar discectomy (PEID), the use of percutaneous endoscopic lumbar discectomy (PELD) for RLDH treatment has increased (Yoshikane et al., 2021; Gadjradj et al., 2022). However, concerns remain regarding potentially less satisfactory outcomes and approach-related complications associated with repeated discectomy (Shin et al., 2011). For instance, scar tissue increases the risk of epidural tears or nerve injury, posing challenges to repeated discectomy. PETD is a more commonly used approach for RLDH treatment than PEID (Kapetanakis, Gkantsinikoudis & Charitoudis, 2019). However, compared with the transforaminal approach for lumbar discectomy, the interlaminar approach has the advantages of good operation habits, a highly efficient operation process, high comfort for patients, wide surgical indications, and relatively controllable complications (Yan et al., 2020; Gao et al., 2021). Furthermore, high iliac crest anatomy, highly dissociated disc herniations, and a steep learning curve limit the application of PETD (Yin, Wang & Liu, 2021; Li, Yan & Cong, 2022). The efficacy of PEID for recurrent herniations is unclear, and its outcomes in patients with RLDH have been evaluated only in a few studies (Liu et al., 2020; Ono et al., 2022). Therefore, this study aimed to assess the effectiveness of PEID in treating RLDH and address knowledge gaps regarding its outcomes and viability for RLDH at L4–5 and L5–S1.

## Materials and Methods

**Figure 1:**
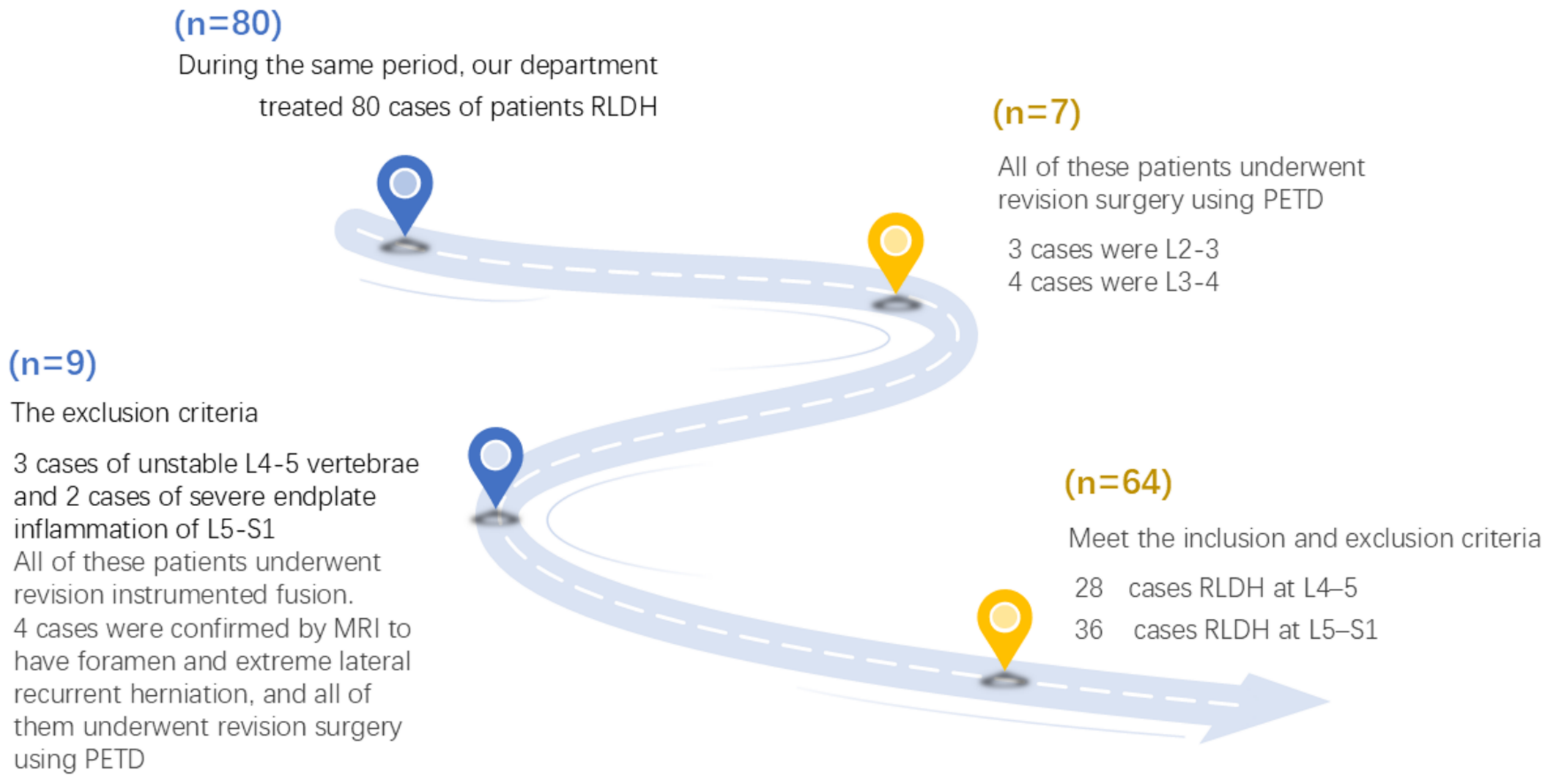
Inclusion and exclusion flow diagram. Abbreviations: RLDH, recurrent lumbar disc herniation; MRI, magnetic resonance imaging; PETD, percutaneous endoscopic transforaminal discectomy.

In this study, we retrospectively analysed the data of 64 consecutive patients who underwent PEID following an initial lumbar discectomy for RLDH. During the same period, our department treated 80 cases of RLDH. Among them, three cases involved L2–3, and four cases involved L3–4. All of these cases underwent PTED revision surgery. Three cases of unstable L4–5 vertebrae and two cases of severe endplate inflammation at L5–S1 were treated with instrumented fusion. Four cases were confirmed to have foramen and extreme lateral recurrent herniation using magnetic resonance imaging (MRI), and all of them underwent PTED revision surgery. Figure 1 shows a CONSORT-style flow diagram of the patient selection process. The study was conducted from June 2022 to December 2024, and 28 and 36 cases of RLDH at L4–5 and L5–S1, respectively, were included. The cohort comprised 44 males and 20 females aged between 26.5 and 75.1 years (mean ± SD: 51.7 ± 11.9 years). The 64 initial discectomy procedures included microendoscopic discectomy (*n* = 14), PELD (*n* = 24), PEID (*n* = 14), and open discectomy (*n* = 12).

The inclusion criteria were: (1) same single-level and ipsilateral side RLDH at L4–5 or L5–S1; (2) RLDH, verified using magnetic resonance imaging (MRI); (3) central type or peripheral central type lesion, confirmed preoperatively using MRI; (4) history of open or other minimally invasive discectomy for patients with RLDH at L4–5 or L5–S1; (5) MRI findings correlating with clinical symptoms and signs, and failed after at least 3 months of conservative treatment.

The exclusion criteria were: (1) segmental instability due to spondylolisthesis, facet joint arthritis or wide decompression performed in initial surgery; (2) spinal infection, tumour or fracture; (3) coexisting psychological diseases or neuromuscular diagnoses, such as depression or Parkinson’s disease; (4) preoperative CT or MRI excluding foramen and extreme lateral recurrent herniation.

This study adhered to the ethical principles outlined in the International Conference on Harmonisation Declaration of Helsinki for Good Clinical Practice (PJ202402). This study was approved by the Institutional Review Board of Dongguan Hospital of Traditional Chinese Medicine. The requirement for informed consent was waived due to the retrospective study design. All revision surgeries were performed by the same team of surgeons, ensuring a standardised approach throughout the trial.

### Surgical procedure

The surgical procedure was conducted with the patient in a prone position under general anaesthesia on a radiolucent bed. Endoscopic interlaminar discectomy was performed as previously described (Yan et al., 2020). A dilator was introduced directly into the bone tissue at the inferior edge of the interlaminar window or lateral facet joint. Subsequently, a cannula sheath with a bevelled opening was directed toward the scar tissue. To protect the dura and nerve tissues, the interlaminar window was expanded to identify and dissect the normal margins of the scar. In all cases, a high-speed drill was used to thin the medial facet lateral to the prior laminotomy before removing a few millimetres of new bone. A description of this surgical technique is reported in detail elsewhere (Liu et al., 2020). This meticulous approach was performed to protect delicate structures while addressing scar tissue and facilitating the subsequent stages of interlaminar discectomy.

### Clinical outcome evaluation

The average operation time was 95.5 min (range, 75–146 min). Blood loss was minimal. Clinical outcomes for back and leg pain were assessed using a visual analogue scale (VAS), with a score range of 1–10. Functional outcomes were measured using the Oswestry Disability Index (ODI) on a 1–100% range. Evaluations were performed preoperatively and at 1 day, 3 months, 12 months, and 2 years postoperatively. VAS and ODI scores were recorded at each interval to identify changes in pain levels and functional ability over time. The final follow-up assessment utilised the MacNab criteria to evaluate the overall outcomes. Based on the MacNab criteria, a successful outcome was defined as excellent or good, indicating positive results in pain relief and functional improvement. This comprehensive approach to outcome evaluation, incorporating subjective measures such as the VAS, ODI, and standardised MacNab criteria, provided a well-rounded assessment of the efficacy and success of PEID for RLDH.

### Statistical analysis

First, after the examination, it was found that preoperative and postoperative VAS and ODI scores were normally distributed at each time point. And then repeated measures analysis of variance (RM-ANOVA) was used to test the overall time effect, and Bonferroni as *post-hoc* tests were employed to compare the differences between pre-op *vs* post-op time points. And the modified MacNab criteria of the final follow-up is to present the group proportions through statistical description. *P*-values < 0.05 were considered statistically significant. Statistical analyses were performed using SPSS Statistics for Windows (Version 20.0; IBM Corp., Armonk, NY, USA)

## Results

This study originally included 64 patients with RLDH at L4–5 and L5–S1. The initial surgery was performed 35.9 ± 26.2 months before the present full endoscopic interlaminar discectomy (FEID) (range: 0.5–108 months). All 64 patients were successfully followed up for at least 24 months, allowing for a comprehensive evaluation of the short-term outcomes and effectiveness of PEID in managing RLDH in the cohort.

**Figure 2:**
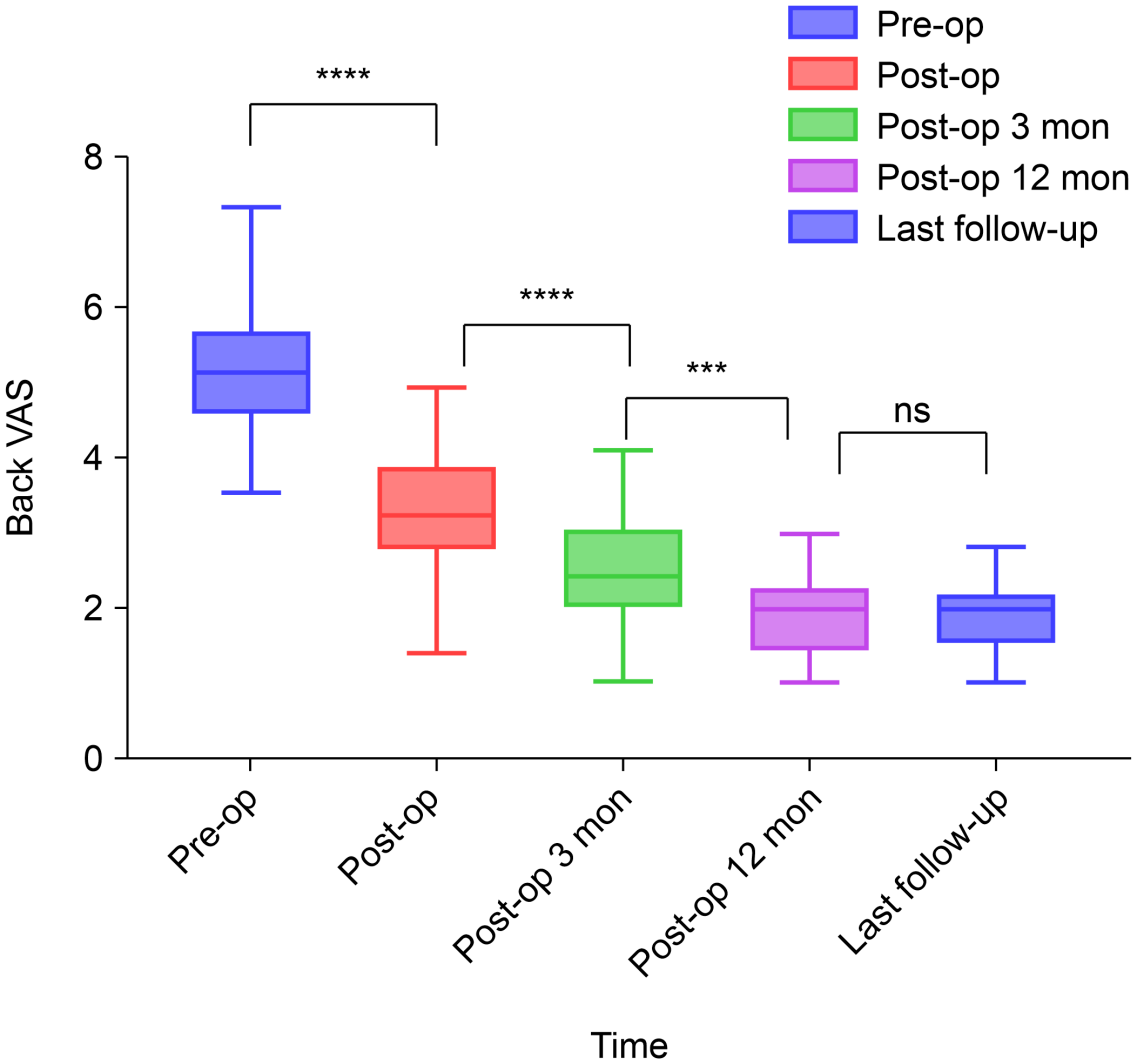
Preoperative and postoperative visual analogue scale scores for back pain. ****, *P* < 0.0001; ***, *P* < 0.001; ns, *P* > 0.05; compared using the unpaired *t*-test. Abbreviations: VAS, visual analogue scale; pre-op, preoperatively; post-op, postoperatively; mon, months; ns, non-significant.

**Figure 3:**
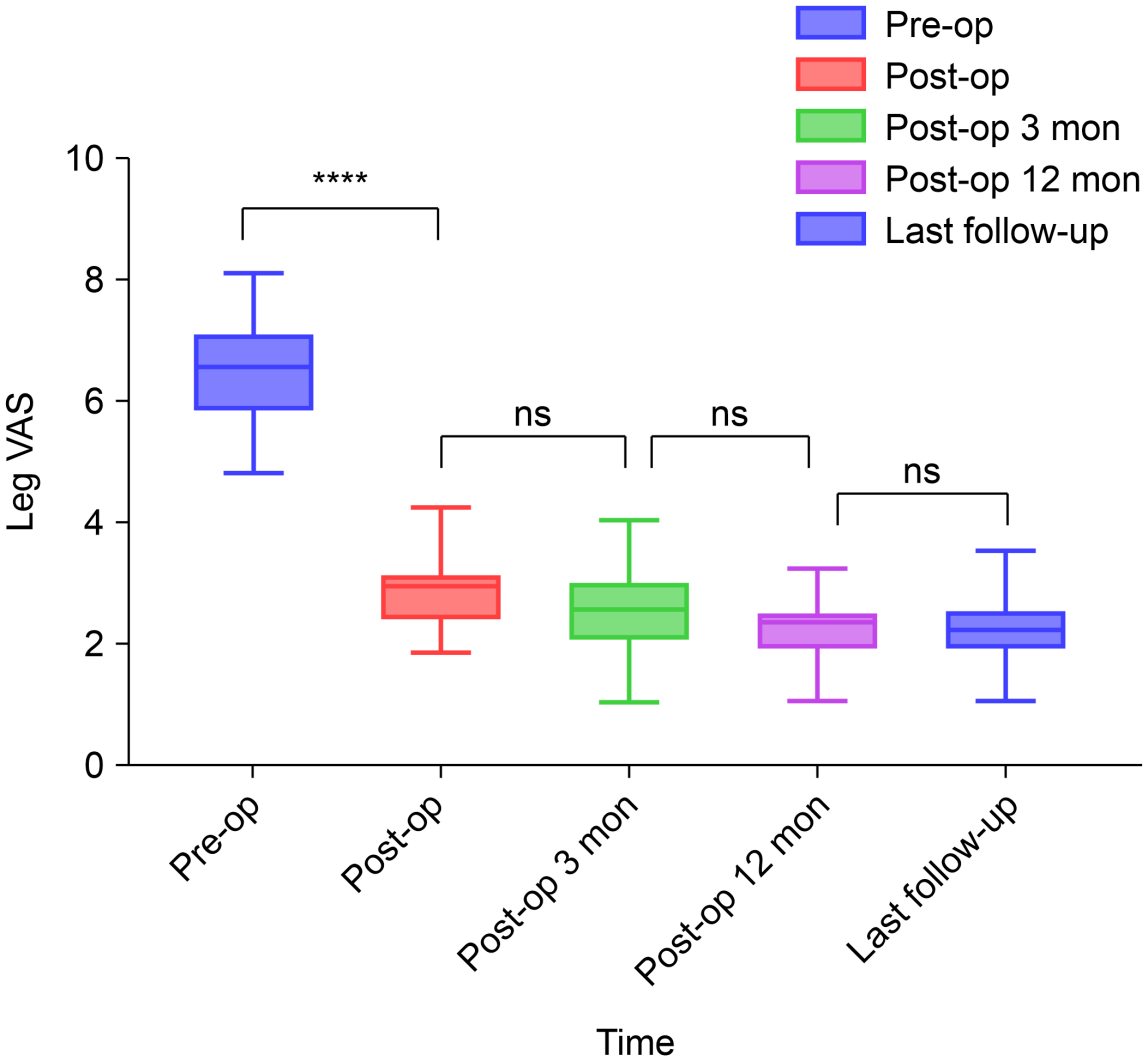
Preoperative and postoperative visual analogue scale scores for leg pain. ****, *P* < 0.0001; ns, *P* > 0.05; compared using repeated measures analysis of variance (RM-ANOVA) used to test the overall time effect, and Bonferroni as *post-hoc* tests employed to compare the differences between pre-op *vs* post-op. Abbreviations: VAS, visual analogue scale; pre-op, preoperatively; post-op, postoperatively; mon, months; ns, non-significant.

The preoperative VAS scores (mean ± standard deviation) for back and leg pain were 5.09 ± 0.86 and 6.43 ± 0.90, respectively. Significant differences in VAS scores for back and leg pain were observed postoperatively, indicating significant improvement (Figs. 2 and 3). These improvements illustrated the substantial positive impact of surgical intervention in alleviating back and leg pain in the study cohort.

**Figure 4:**
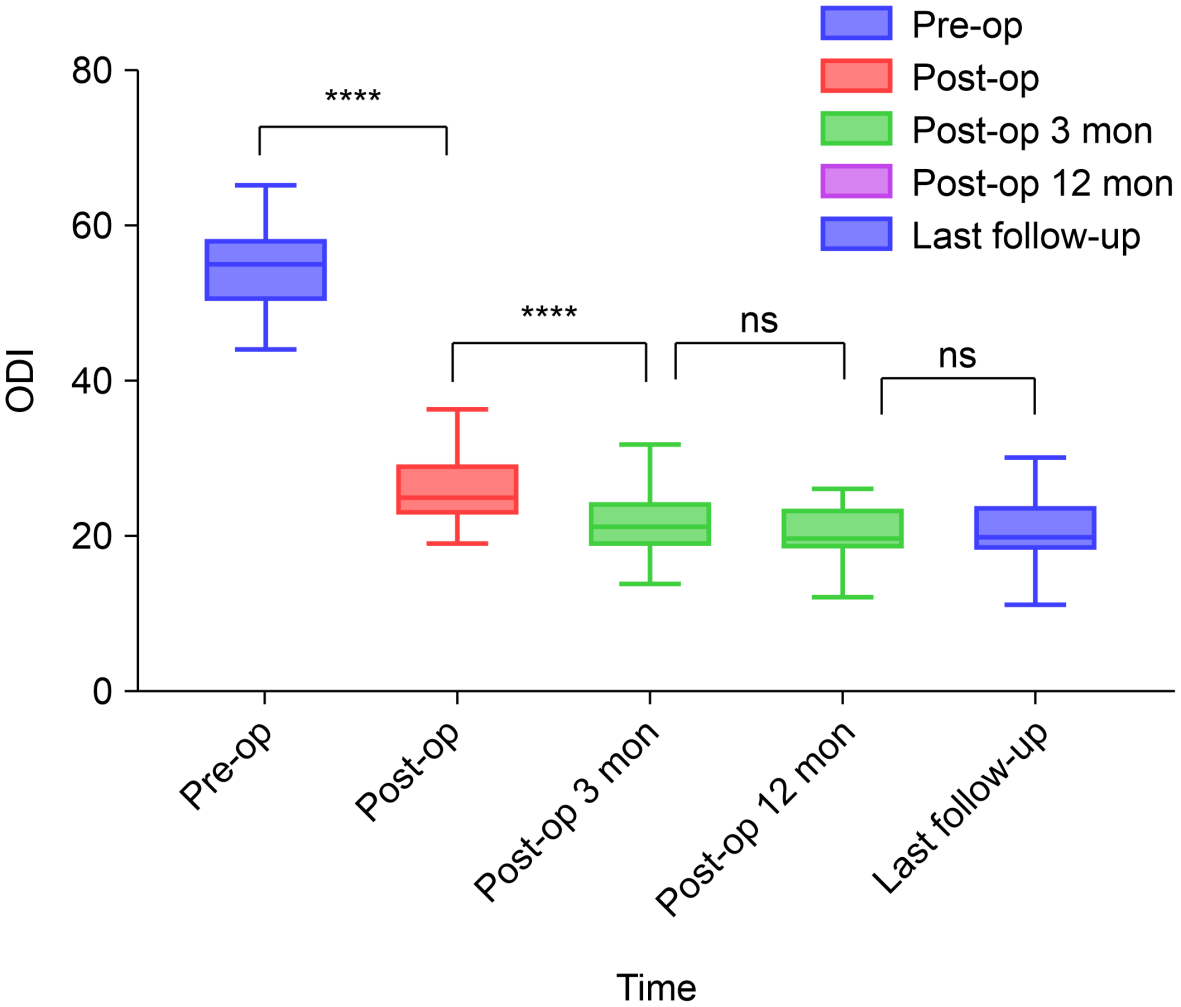
Preoperative and postoperative Oswestry Disability Index scores. ****, *P* < 0.0001; ***, *P* < 0.001; ns, *P* > 0.05; compared using repeated measures analysis of variance (RM-ANOVA) used to test the overall time effect, and Bonferroni as *post-hoc* tests employed to compare the differences between pre-op *vs* post-op. Abbreviations: ODI, Oswestry Disability Index; pre-op, preoperatively; post-op, postoperatively; mon, months; ns, non-significant.

The ODI scores were recorded as 54.51 ± 5.60, 25.45 ± 4.22, 24.41 ± 4.10, 19.96 ± 3.54, and 20.52 ± 4.49 at each respective time point. A significant improvement in ODI scores was observed immediately after surgery and was sustained at the 3-month follow-up. Although a slight increase in ODI score was observed at the last follow-up, this difference was not significant, as shown in Fig. 4. The stability of ODI scores over time underscores the enduring positive impact of surgical intervention on functional disability, reinforcing the sustained benefits observed in the cohort.

**Figure 5:**
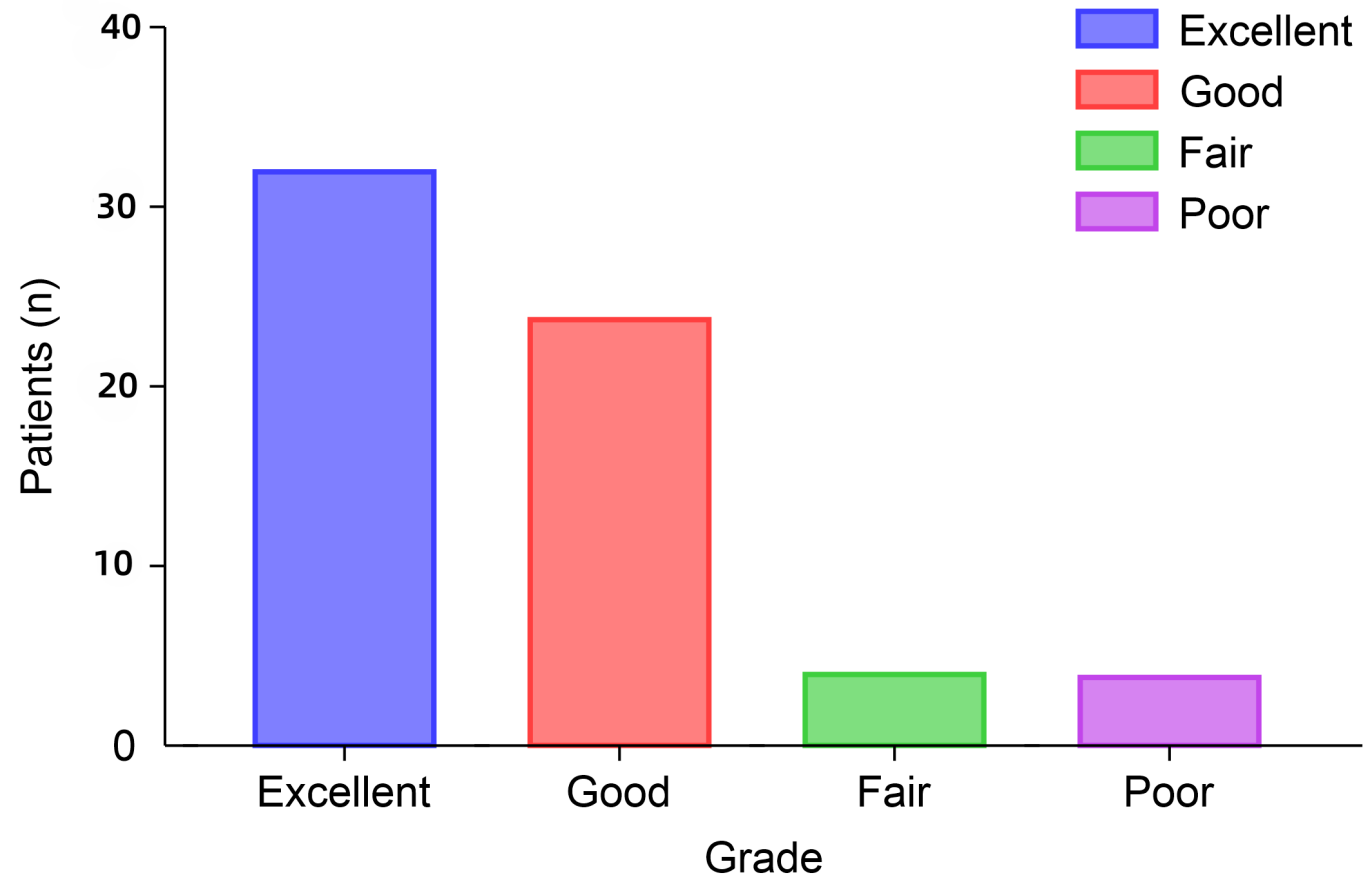
Modified MacNab criteria at the last follow-up. n, number. The modified MacNab criteria of the final follow-up is to present the group proportions through statistical description.

According to the modified MacNab criteria, the clinical prognosis of PEID revision yielded a favourable outcome, with an 88% rate of excellent and good outcomes. The distribution of outcomes among the patients was as follows: excellent in 32 patients, good in 24, fair in four, and poor in four (Fig. 5). This distribution reflects a predominantly positive clinical prognosis, highlighting the effectiveness of PEID revision in achieving satisfactory outcomes in most patients in the study cohort. Throughout the entire clinical study, no cases were lost to follow-up.

### Complications

**Figure 6:**
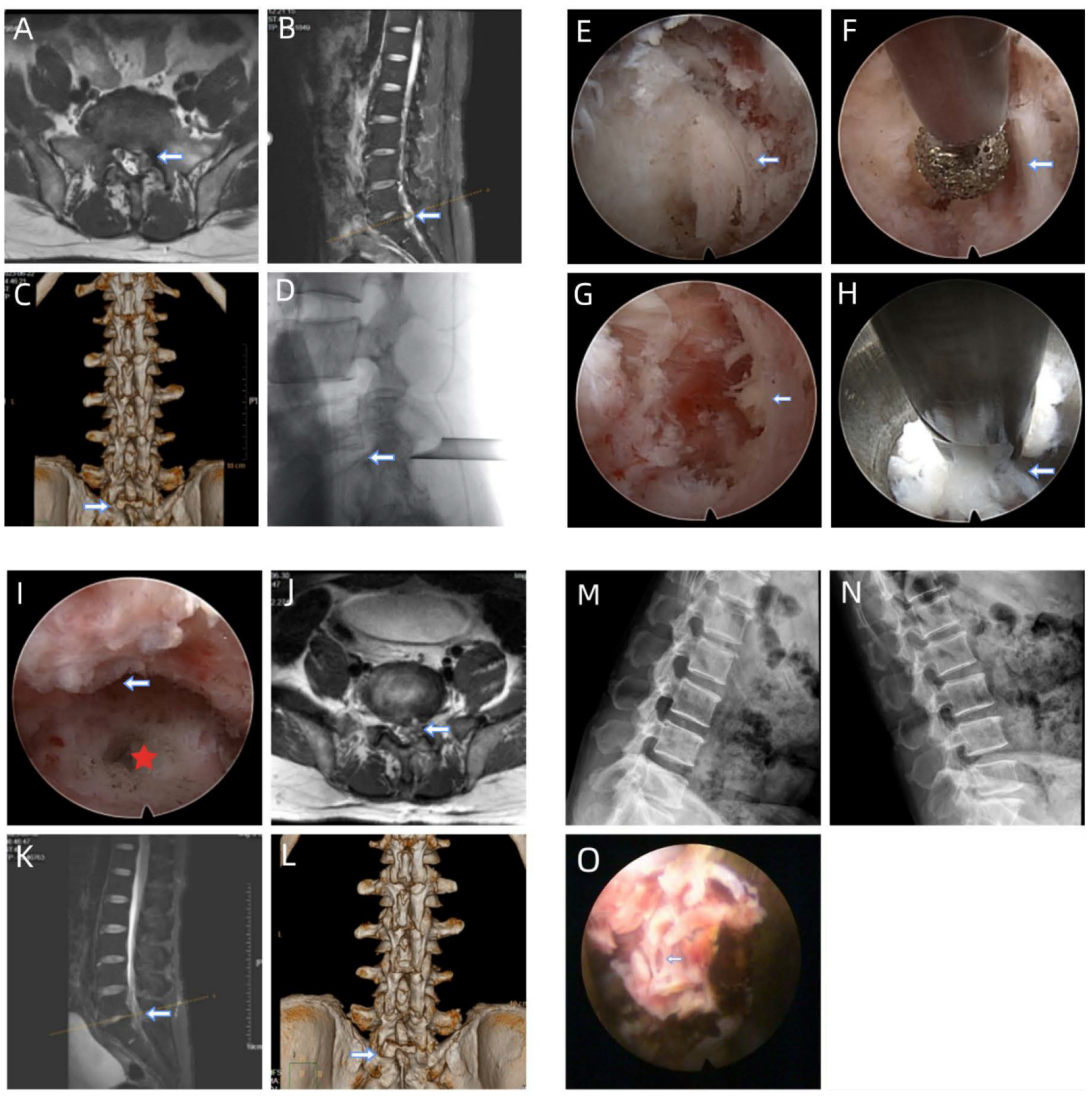
Illustrative study case. The patient, a 51-year-old male, presented with recurrent left leg pain and a history of open lumbar microdiscectomy at L5–S1 9 years prior. (A) and (B): Preoperative T2-weighted axial and sagittal magnetic resonance images showing the condition before surgery. (C): Preoperative three-dimensional computed tomography scan showing the primitive superior and inferior lamina and medial facet joint. (D): The positioning of the external cannula is confirmed intraoperatively. (E): The working sheath is docked on the lateral osseous margin of the interlaminar window. (F): The facet and inferior lamina are partially removed using a high-speed drill to create adequate working space and further separate soft tissue from osseous structures. (G): The ligamentum flavum is excised from fresh tissue. (H): The disc material is successfully removed. (I): Satisfactory release of nerve compression is observed under endoscopy. (J) and (K): Postoperative axial and sagittal magnetic resonance images show effective removal of the disc material. (L): Three-dimensional computed tomography scan showing enlargement of the superior and inferior lamina and medial facet joint with preserved articular process stability. (M) and (N): Dynamic radiograph showing no postoperative lumbar instability at the last follow-up. (O): Epidural tear occurred, and repair was not performed.

**Figure 7:**
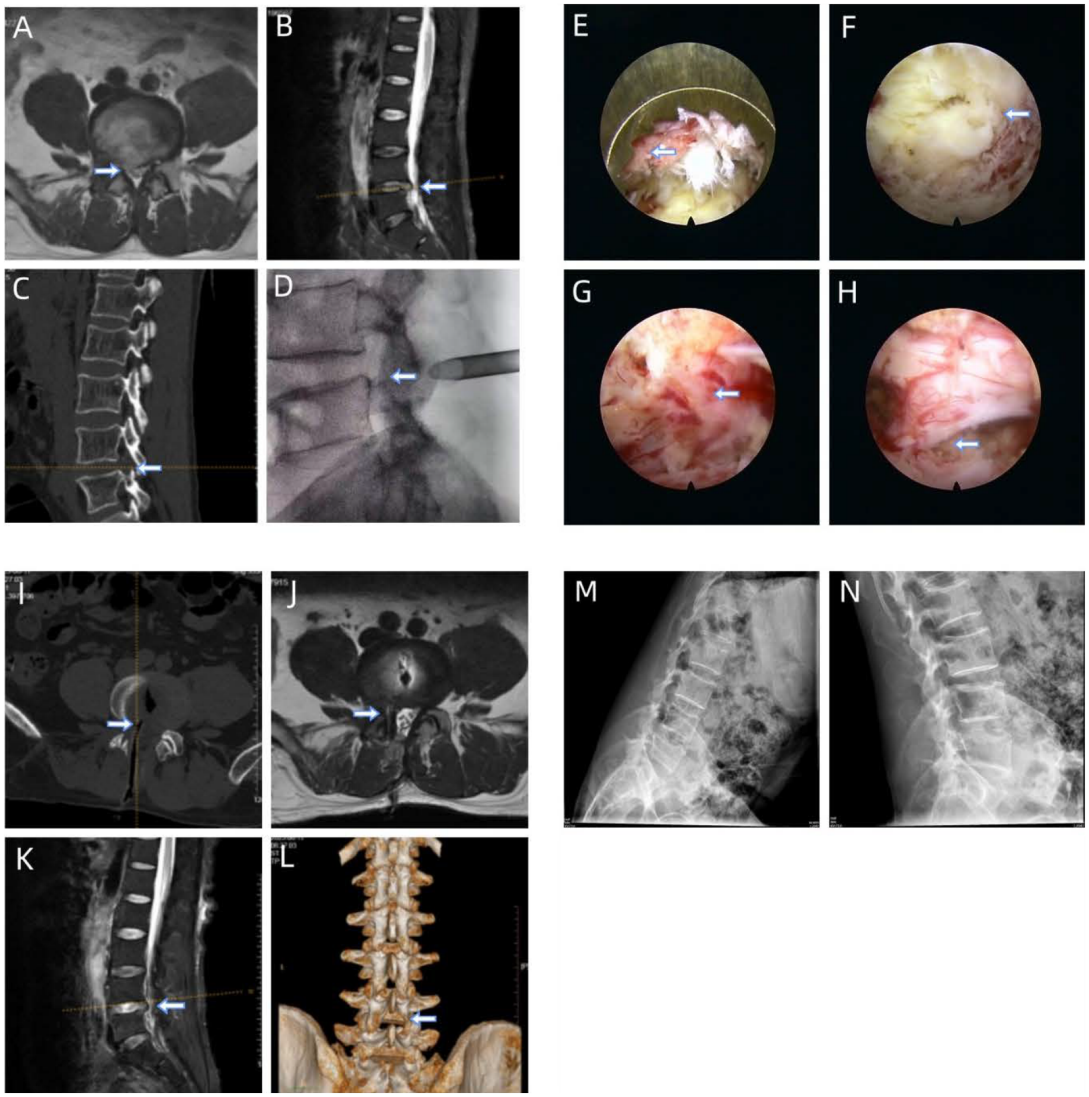
Illustrative study case. The patient, a 47-year-old male, complained of recurrent right leg pain at L4–5. The primary surgery was performed using PETD 1 year prior. (A) and (B) Preoperative T2-weighted axial and sagittal magnetic resonance images showing the patient’s condition before surgery. (C) The superior facet of L5 is removed during the initial operation. (D) The position of the external cannula is confirmed during surgery. (E) The facet bone is routinely removed using a trephine through a posterior approach. (F) Local ligamentum flavum. (G) Dura mater and scar tissue. (H) Complementarily released nerve tissue is observed on endoscopy. (I) A drainage tube is positioned too deep, irritating the nerve root. (J) and (K) Postoperative magnetic resonance images reveal effective removal of the disc material. (L) Three-dimensional computed tomography scan showing enlargement of the superior and inferior laminae and medial facet joint with preserved articular process stability. (M) and (N) Dynamic radiographs showing no postoperative lumbar instability at the last follow-up.

Four cases of nerve root injury were observed, each involving a laceration of the outer nerve membrane less than three mm. No wound infection cases were observed. A small epidural tear was identified in one patient (Fig. 6O); however, suturing was unnecessary. Four cases of postoperative dysaesthesia were reported. In one case, the drainage tube was placed too deeply after surgery, causing nerve root irritation; however, the symptoms resolved after the tube was removed (Fig. 7I). The other cases were suspected to result from radiofrequency thermal injury; the symptoms resolved 3 months postoperatively with the implementation of acupuncture and physiotherapy. Two patients recurred with disc herniation 1 year after revision; one underwent posterior PEID revision again, and the other required lumbar fusion due to secondary lumbar segmental instability. Both cases involved patients with diabetes, and their postoperative rest period was less than 2 weeks. These findings underscore the overall safety profile of the PEID procedure in the context of this study. All the details of the complications can be found in Table 1.

## Discussion

Considering that conventional open discectomy is the standard surgical procedure for treating RLDH, the management of RLDH remains controversial. Some studies have concluded that secondary conventional revision discectomy can effectively and safely manage RLDH (Ahsan et al., 2020). Guan et al. (2017) reported that repeat discectomy and instrumented fusion yielded comparable clinical outcomes in patients with recurrent disc herniation. The patients in the repeat discectomy group exhibited significantly shorter operative times, shorter hospital stays, lower hospital costs, and a lower likelihood of requiring postoperative acute rehabilitation. Repeat discectomy is the preferred surgical treatment for RLDH without objective evidence of spinal instability (Hlubek & Mundis, 2017), with PETD and PEID being alternative options. In a study on percutaneous endoscopic discectomy for recurrent disc herniation, 81% of patients exhibited a favourable outcome (Kim et al., 2014). Furthermore, in a previous study, the rate of excellent and good outcomes among patients who underwent PETD for RLDH was 83.9% at the 2-year postoperative follow-up (Xu et al., 2023a; Xu et al., 2023b).

PETD offers several distinct advantages as a minimally invasive surgical procedure (Arif et al., 2020; Yoshikane et al., 2021; Wang et al., 2023). Typically performed under local anaesthesia, normal paraspinal structures are preserved during this procedure, resulting in minimal postoperative pain and a shorter hospital stay (Arif et al., 2020). Wang et al. (2023) treated 68 patients with RLDH using fenestrated endoscopic discectomy, with 88.23% of the patients exhibiting excellent or good outcomes based on the modified MacNab criteria.

Furthermore, PEID offers advantages such as avoiding an iliac crest block and enabling rapid positioning puncture. PEID is also associated with reduced intraoperative radiation exposure. Liu et al. (2020) reported performing full endoscopic interlaminar discectomy (FEID) in 24 consecutive patients with RLDH; significant improvements were observed in the VAS scores for leg and back pain and the ODI scores after treatment. In a systematic review and meta-analysis, PEID was superior to PETD in terms of fluoroscopy time and operative duration, suggesting that PEID may be the preferred surgical option for lumbar disc herniation at L5–S1 (Chen et al., 2018). Finally, PEID typically results in fewer residual symptoms of low back pain compared with PETD (Xu et al., 2023a; Xu et al., 2023b). In our cohort, the use of PEID demonstrated comparable outcomes in alleviating back and leg pain and improving functional status. These findings are consistent with those of previous studies (Liu et al., 2020; Li et al., 2024).

Approach-related complications are a significant concern in repeat surgeries for RLDH, which are often associated with challenges posed by epidural adhesions and scar tissue (Yoshikane et al., 2021). Dower et al. (2016) reported a slightly higher rate of incidental durotomies in the minimally invasive discectomy group (5.67%) than in the conventional open discectomy group (2.90%) (relative risk: 2.05; 95% confidence interval: 1.05–3.98). Mehren, Wanke-Jellinek & Korge (2020) observed a 1.5% (11/894) rate of initial lumbar disc surgeries and a 7.7% (9/117) rate of dural lesions. In a meta-analysis evaluating surgical outcomes after endoscopic or conventional discectomy for RLDH, endoscopic discectomy was associated with a significantly shorter operative time and a lower risk of complications (Wang et al., 2023). However, Liu et al. (2020) reported a small durotomy in two patients with no visible cerebrospinal fluid leakage. Chen et al. (2015) found no intraoperative incidental durotomies in the PELD group, highlighting the challenges posed by epidural adhesion and scar tissue. In a study by Wang et al. (2023) on fenestrated endoscopic discectomy for RLDH, no complications such as dural tears, nerve root injury, infection, or haematoma were observed in 68 cases. In a meta-analysis investigating complications associated with PELD for RLDH, Zhao et al. (2022) observed lower overall complication and dural tear rates in the PELD group than in the traditional fenestration nucleus pulposus removal group (Li, Yan & Cong, 2022). In this study, an epidural tear occurred in one patient, and repair was not performed owing to its small size (<two mm) and the absence of visible postoperative cerebrospinal fluid leakage; further intervention was deemed unnecessary. Even if a dural tear occurs during surgery, the risk of cerebrospinal fluid leakage is minimal because the muscle channel collapses after device withdrawal (Li et al., 2016).

The time of recurrence, the positional relationship between the recurrent disc and nerve root, and the surgeon’s technical mastery and preference must be considered when selecting a surgical approach. Ono et al. (2022) suggested that fenestrated endoscopic discectomy with interlaminar access for RLDH is best performed within 2 weeks before adhesion progression, whereas late-stage revisions (>3 weeks) are often performed at the surgeon’s discretion. Wang et al. (2023) reported that both FEID and FETD were effective and safe for RLDH at >2 weeks; however, FEID appeared more efficient for RLDH at L4–5 after a previous FETD and RLDH at L5–S1. Choi et al. (2013) demonstrated that transforaminal PELD is preferred for shoulder-type, centrally located, and recurrent disc herniation, whereas interlaminar PELD is preferred for axillary-type and migrated discs, particularly those of high grade. Li et al. (2024) reported that PEID was superior to PETD in reducing operative time and frequency of fluoroscopy. The interlaminar approach of FEID more closely resembles traditional open discectomy. Therefore, we selected FEID for RLDH treatment.

**Table 1:** Complications.

Type	Number of cases	Management	Outcome
Nerve root injury	four cases	clinical observation	recover
Postoperative dysaesthesia	one case drainage tube irritation three cases radiofrequency thermal injury	remove the drainage tube three cases clinical observation	recover
Recurred with disc herniation	two cases	one case revision one case lumbar fusion	recover

To our knowledge, numerous factors influence recurrent disc herniation, including intervertebral disc degeneration, endplate changes, surgical techniques, and patient clinical characteristics (age, gender, body mass index, duration of symptoms, type of protrusion, smoking status, and diabetes). According to an original study by Yaman et al. (2017) patients with RLDH had higher intervertebral disc height and higher body mass index before surgery. The Modic endplate changes were associated with a higher tendency for lumbar disc herniation recurrence. In our cases, two patients experienced a recurrence of intervertebral disc protrusion 1 year after the surgery; both of these patients had diabetes, and the postoperative rest period for both was less than 2 weeks. Special procedures associated with scar tissue dissection are as follows: (I) separate the scar tissue from the bony margin use a dissector or curette, with additional lamina or facet resection, using a high-speed drill in adhesive cases; (II) dissect the epidural scar tissue cranially, caudally, and somewhat medially to widen the plane, then move the working sheath forward and rotate to further separate the scar tissue and detect disc fragments; (III) Dissect the scar tissue from the medial facet joint rather than from neural tissue, and retract the scar and neural tissues together to avoid dural tears (Kim et al., 2012).

Regarding postoperative spinal stability, transforaminal discectomy has advantages over posterior discectomy in preserving paraspinal and posterior spinal structures, including the laminae, facet joints, ligaments, and muscles (Xu et al., 2023a; Xu et al., 2023b). According to a comparative study by Lee et al. (2009) in which cadavers were used, one patient (3.4%) exhibited newly developed instability after repeated open lumbar microdiscectomy, whereas no patient exhibited instability after PELD at the final follow-up. Compared with conventional procedures, endoscopic methods can minimise tissue trauma; additionally, in most cases, an osseous margin of <4 mm is required for removal, and spinal stability can be preserved (Liu et al., 2020). In our cohort, one case required lumbar fusion surgery owing to recurrent intervertebral disc protrusion and secondary segmental instability of the lumbar spine, possibly because of the minimal destruction of the posterolateral structure and the relatively short follow-up period.

### Limitations

This study had some limitations, including its retrospective design, small sample sizes, and short follow-up period. No subgroup analysis was conducted regarding the preoperative Modic types of the endplates and other confounding factors such as diabetes and peripheral neuropathy. These limitations may affect the generalisability of our findings; therefore, caution should be exercised when applying our findings to broader populations.

## Conclusions

PEID achieves good clinical efficacy in the treatment of RLDH. PEID can be regarded as an effective reference method for treating patients with RLDH at L4–5 and L5–S1.

## Supplemental Information

10.7717/peerj.21081/supp-1 Supplemental Information 1 Back pain VAS

10.7717/peerj.21081/supp-2 Supplemental Information 2 Leg pain VAS

10.7717/peerj.21081/supp-3 Supplemental Information 3 ODI score

10.7717/peerj.21081/supp-4 Supplemental Information 4 MacNab criteria

## Additional Information and Declarations

### Data Availability

The following information was supplied regarding data availability: The raw data are available in the Supplemental Files.
